# Systemic Molecular Alterations of *TP53*, *SIRT-1*, and miR-34a Expression in Atrial Fibrillation: A Prospective Exploratory Biomarker Study

**DOI:** 10.3390/ijms27125633

**Published:** 2026-06-22

**Authors:** Monika Różycka-Kosmalska, Izabela Szymczak-Pajor, Agnieszka Śliwińska, Małgorzata Kozłowska, Jerzy Krzysztof Wranicz, Marcin Kosmalski

**Affiliations:** 1 Department of Electrocardiology, Medical University of Lodz, 92-213 Lodz, Poland; monika.rozycka-kosmalska@umed.lodz.pl (M.R.-K.); jerzy.wranicz@umed.lodz.pl (J.K.W.); 2 Department of Nucleic Acid Biochemistry, Medical University of Lodz, 92-213 Lodz, Poland; izabela.szymczak@umed.lodz.pl (I.S.-P.); agnieszka.sliwinska@umed.lodz.pl (A.Ś.); malgorzata.kozlowska@umed.lodz.pl (M.K.); 3 Department of Clinical Pharmacology, Medical University of Lodz, 90-153 Lodz, Poland

**Keywords:** atrial fibrillation, TP53, SIRT-1, miR-34a, atrial remodeling

## Abstract

p53, miR-34a, and SIRT-1 are involved in cellular stress responses, senescence, and inflammation—processes central to the pathophysiology of atrial fibrillation (AF). In this study, circulating *TP53* and *SIRT-1* serum miR-34a expression were determined in patients with and without AF, in order to assess their associations with AF. We also checked their potential diagnostic utility as systemic biomarkers associated with AF. The study included 189 adults, 94 AF+, 95 AF−. Clinical, anthropometric, and biochemical data were collected. Whole-blood *TP53* and *SIRT-1* mRNA expression and serum miR-34a expression were quantified by RT-qPCR. ROC analysis and Youden-derived odds ratios assessed exploratory diagnostic performance. AF patients had significantly higher expression of *TP53* (0.0352 vs. 0.0253; *p* < 0.001) and miR-34a (0.0215 vs. 0.0099; *p* < 0.001), but significantly lower expression of *SIRT-1* (0.0079 vs. 0.0145; *p* < 0.001). The level of *SIRT-1* expression showed the highest discriminatory performance (exploratory AUC = 0.6987; *p* < 0.0001). *TP53* expression levels exceeding 0.0295 were associated with nearly threefold higher odds of AF (OR = 2.92, 95% CI: 1.61–5.28, *p* = 0.0006), whereas the expression levels of *SIRT-1* and miR-34a were not significantly associated with AF in cut-off analysis. In the AF group, a positive correlation was found between the expression of *TP53* and *SIRT-1* (*Rho* = 0.3609, *p* < 0.001); however, it was not consistent with a canonical model of miR-34a-mediated *SIRT-1* suppression. In turn, the expression of miR-34a correlated positively with age and C-reactive protein level and negatively with estimated glomerular filtration rate (eGFR). The obtained results suggest that AF is associated with altered expression of circulating *TP53*, *SIRT-1*, and miR-34a. However, due to the fact that the expression levels were measured in peripheral compartments, and not in atrial tissue, the obtained results should not be interpreted as direct evidence of AF-related atrial remodeling. For these reasons, further investigations involving simultaneous measurements of the TP53/miR-34a/SIRT-1 regulatory axis, both in the circulating compartment and atrial tissue, should be performed.

## 1. Introduction

Atrial fibrillation (AF) is one of the most common cardiac arrhythmias, classified as a supraventricular arrhythmia characterized by disorganized atrial electrical activity leading to ineffective atrial contraction. On electrocardiography (ECG), AF is identified by the absence of distinct P waves and an irregular ventricular rhythm [1].

The escalating global incidence, prevalence, and lifetime risk of AF are primarily attributed to demographic aging, enhanced diagnostic detection, and improved survival rates among patients with pre-existing cardiovascular diseases. The distribution of AF varies significantly across geographical regions and is heavily stratified by age and sex. Risk factors for AF can be broadly divided into non-modifiable and modifiable factors. Non-modifiable risk factors include advanced age, sex, taller stature, and genetic predispositions including variants in ion channel, structural, signaling, and transcription factor genes. Modifiable risk factors include smoking, increased body weight, elevated blood pressure, diabetes, and cardiovascular diseases such as heart failure or past myocardial infarction. Additional modifiable factors include excessive alcohol consumption, sleep apnea, hyperthyroidism, and physical inactivity. AF is also associated with an increased risk of stroke, heart failure, myocardial infarction, chronic kidney disease, dementia, and all-cause mortality [2,3].

The pathophysiology of AF is inherently complex and multifactorial, characterized by an interplay of structural, electrical, and metabolic atrial remodeling. These pathological alterations are frequently driven by intracellular Ca^2+^ disturbances, metabolic dysregulation, autonomic imbalance, hormonal changes, inflammation, and oxidative stress [4,5]. In recent years, increasing attention has been devoted to molecular pathways associated with the DNA damage response and epigenetic regulation, including the potential involvement of TP53 and its related regulatory molecules, such as miR-34a and SIRT-1, in the development of AF [6,7,8].

p53 serves as a sequence-specific DNA-binding transcription factor that regulates the expression of genes involved in cell cycle control, apoptosis, DNA repair, metabolism, and immune responses under cellular stress conditions such as DNA damage, oxidative stress, or nutrient deprivation. In addition, p53 influences cellular energy metabolism by suppressing glycolysis and promoting mitochondrial respiration, while also regulating the pathways related to antioxidant defense, mitochondrial quality control, and autophagy [9,10]. Emerging evidence suggests that increased p53 activity may contribute to the development and progression of AF [11,12].

MicroRNAs (miRNAs) are a class of short, endogenous non-coding RNA molecules that act as negative regulators of gene expression. They primarily exert their effects through post-transcriptional mechanisms such as mRNA degradation or inhibition of translation. Through these mechanisms, they regulate numerous biological processes, including apoptosis, cell proliferation, immune responses, aging, differentiation, autophagy, metabolic pathways, and cellular structural organization. Dysregulation of miRNAs has been implicated in the pathogenesis of various cancers, neurological disorders, obesity, and cardiovascular diseases [13,14].

Among them, the miR-34 family is one of the most conserved groups of microRNAs and plays an important role in the p53 regulatory network [15]. Importantly, miR-34a participates in a canonical regulatory feedback loop with p53, and increased miR-34a expression may suppress p53 signaling through post-transcriptional mechanisms, indicating an inverse regulatory relationship between these molecules [16]. Available data indicate that miR-34a is associated not only with various types of cancer, metabolic disorders, and neurodegenerative diseases but also with the aging process of the cardiovascular system [17,18,19,20,21,22]. Increased miR-34a expression has been linked to cardiac aging, myocardial remodeling, and endothelial dysfunction, partly through the regulation of SIRT-1 and pathways related to apoptosis and oxidative stress [23,24,25]. Moreover, metabolic conditions and certain pharmacological agents may modulate miR-34a expression, suggesting its potential role as both a biomarker and a therapeutic target [26]. There are also reports linking miR-34a expression with AF [7].

SIRT-1 belongs to the sirtuin family of NAD^+^-dependent deacetylases that remove acetyl groups from lysine residues of target proteins. Importantly, sirtuins also deacetylate lysine residues in histone proteins, thereby influencing chromatin structure and regulating gene expression. Through this epigenetic mechanism, sirtuins control multiple cellular processes, including DNA repair, metabolic homeostasis, stress resistance, and aging [27,28]. SIRT-1 plays a key role in energy metabolism in the heart and other organs, such as the liver, and modulates various biological processes including cell survival, oxidative stress, inflammation, and aging [29]. SIRT-1 also regulates p53-dependent apoptosis by deacetylating p53 and modulating its transcriptional activity. *SIRT-1* has been shown to be inhibited by miR-34a, thereby contributing to the activation of apoptosis [30]. It has been suggested that SIRT-1 may exert a protective effect against the development of AF [8,31].

Experimental studies have shown functional interactions among p53, miR-34a, and SIRT-1 in cellular stress responses. In this pathway, p53 transcriptionally activates miR-34a, miR-34a suppresses *SIRT-1* expression, and SIRT-1 may modulate p53 activity through deacetylation [30]. These interactions form a positive feedback loop linked to apoptosis, senescence, oxidative stress, and cardiovascular aging [22,32,33]. Although p53, miR-34a, and SIRT-1 have each been implicated in cardiovascular stress responses and AF-related biology, evidence that they act as a coordinated circulating regulatory loop in AF is lacking. Therefore, the aim of this study was to evaluate whole-blood *TP53* and *SIRT-1* mRNA expression and serum miR-34a expression levels in patients with and without AF, in order to assess their associations with clinical and biochemical parameters, and to explore their potential diagnostic utility as systemic biomarkers associated with AF.

## 2. Results

Table 1 presents comparisons of the prevalence of selected comorbidities and medications used by participants in AF+ group and those without AF (AF− group). Patients in the AF+ group showed a significantly higher prevalence of nicotine use compared with AF− individuals.

The anthropometric characteristics and BP of the studied groups are presented in Table 2. The AF+ group did not differ from the control group (AF−) in terms of age, sex, anthropometric parameters, SBP, DBP, or ejection fraction (EF).

As shown in Table 3, the AF+ group and AF− (control) group did not differ significantly regarding eGFR, FPG, HbA1c, uric acid, total cholesterol, LDL-CH, HDL-CH, TG, ALT, AST, GGTP, total bilirubin, or CRP levels. In contrast, urea and creatinine levels were significantly higher in the AF+ group.

**Figure 1:**
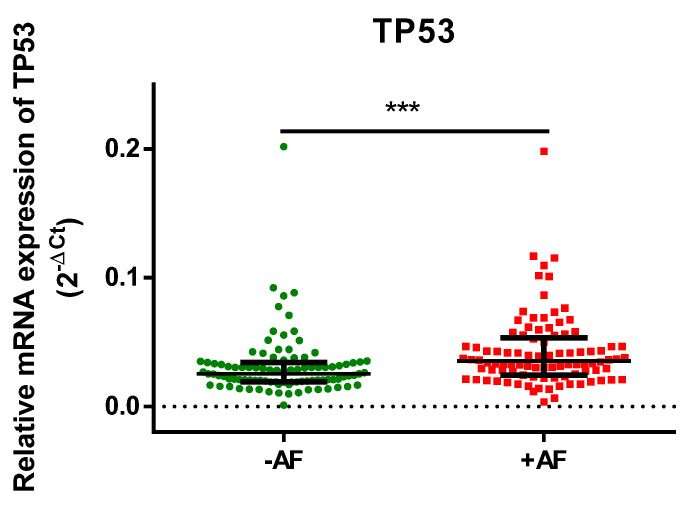
Boxplot of *TP53* expression level in the control (AF−) and AF (AF+) groups. Relative *TP53* expression was quantified by RT-qPCR. Data distributions are presented as box-and-whisker plots representing medians with upper and lower quartiles. Middle line, median; box, interquartile range; whisker, range (including outliers). *** *p* < 0.001 compared to AF− (control) group.

*TP53* mRNA expression was significantly higher in the AF+ group than in the AF− group (0.0352 vs. 0.0253; *p* < 0.001; Figure 1).

**Figure 2:**
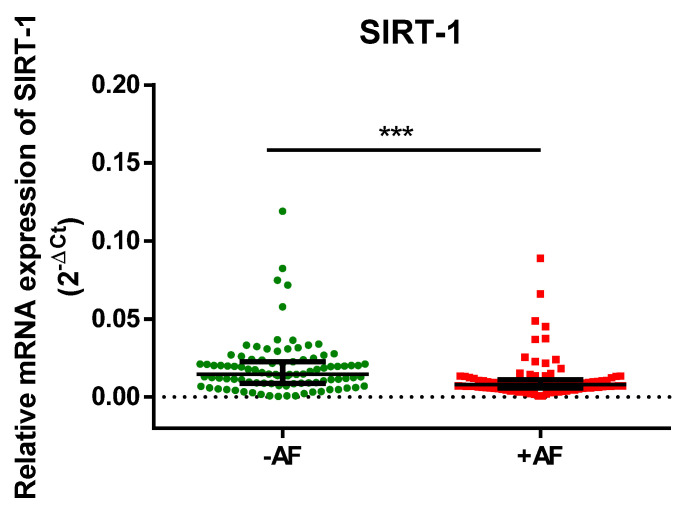
Boxplot of *SIRT-1* expression level in the control (AF−) and AF (AF+) groups. Relative *SIRT-1* expression was quantified by RT-qPCR. Data distributions are presented as box-and-whisker plots representing medians with upper and lower quartiles. Middle line, median; box, interquartile range; whisker, range (including outliers). *** *p* < 0.001 compared to control group.

In contrast to *TP53* expression, *SIRT-1* expression was significantly lower in the AF+ group than in the AF− group (0.0079 vs. 0.0145; *p* < 0.001; Figure 2).

**Figure 3:**
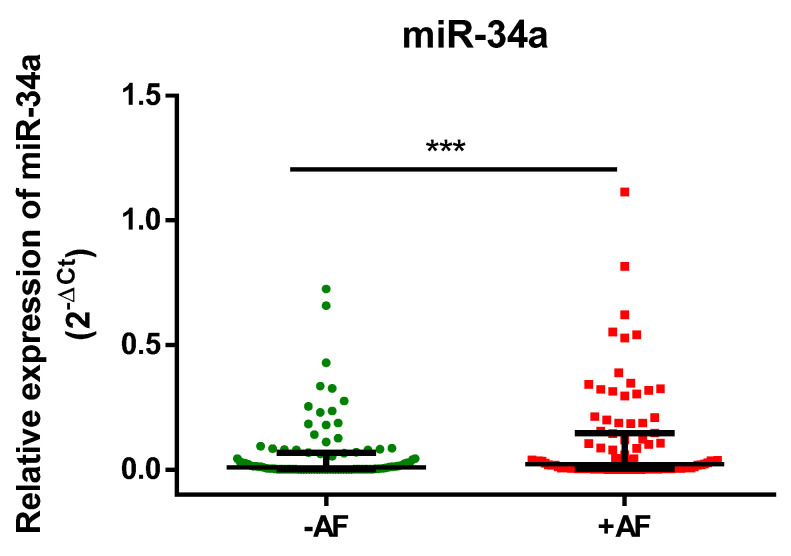
Boxplot of serum level of miR-34a expression in the control (AF−) and AF (AF+) groups. miR34-a expression was quantified by RT-qPCR. Data distributions are presented as box-and-whisker plots representing medians with upper and lower quartiles. Middle line, median; box, interquartile range; whisker, range (including outliers). *** *p* < 0.001 compared to control group.

Serum miR-34a expression was significantly higher in the AF+ group than in the AF− group (0.0215 vs. 0.0099; *p* < 0.001; Figure 3).

Table 4 presents the results of the univariate correlation analysis between *TP53* expression and the patients’ clinical parameters. In the AF+ group, *TP53* expression was moderately positively correlated with *SIRT-1* expression (*Rho* = 0.3609, *p* < 0.001), whereas no significant correlation was observed between *TP53* expression and serum miR-34a levels. The inverse correlation between serum miR-34a and *SIRT-1* expression was observed only in the AF− group and was not present in AF patients. Therefore, the correlation analyses did not show a consistent circulating TP53/miR-34a/SIRT-1 pattern in the AF group.

Table 5 summarizes the results of the univariate correlation analysis between *SIRT-1* expression and the patients’ clinical parameters. In the AF+ group, *SIRT-1* expression was moderately positively correlated with *TP53* expression (*Rho* = 0.3609, *p* = 0.0003), T-CH (*Rho* = 0.2152, *p* = 0.0371), and TG (*Rho* = 0.2601, *p* = 0.0113). In the AF− group, *SIRT-1* expression was positively correlated only with HDL-CH (*Rho* = 0.2116, *p* = 0.0394). No significant correlations were found between *SIRT-1* expression and the other anthropometric or biochemical parameters analyzed.

Table 6 presents the results of the univariate correlation analysis between serum miR-34a levels and the clinical parameters of the patients. In the control group, serum miR-34a levels were positively correlated with age (*Rho* = 0.3999, *p* < 0.001) and TG levels (*Rho* = 0.2951, *p* = 0.0036), and negatively correlated with EF (Rho = −0.2690, *p* = 0.0083), LDL-CH (*Rho* = −0.2700, *p* = 0.0081), and *SIRT-1* expression (*Rho* = −0.2850, *p* = 0.0051).

**Figure 4:**
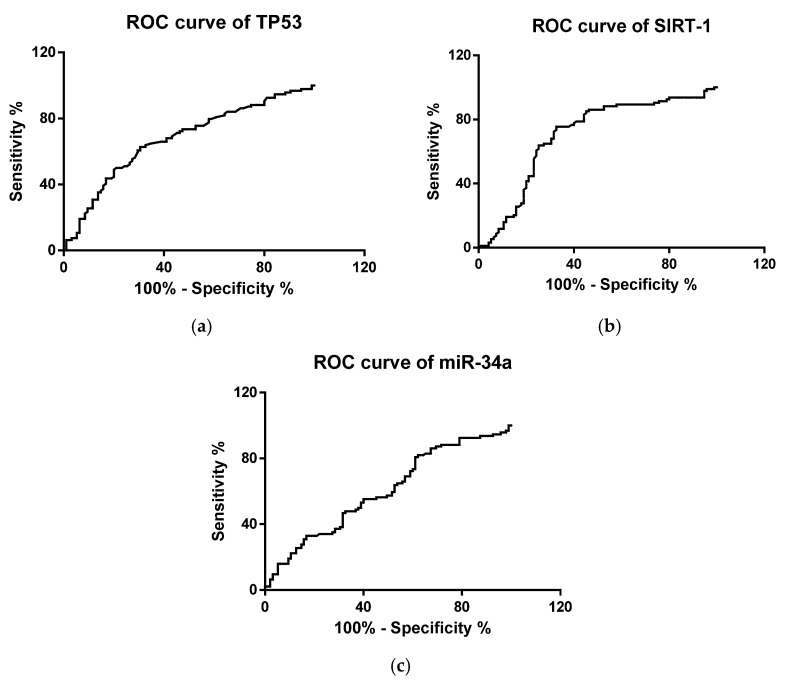
Receiver operating characteristic (ROC) curves for the analyzed biomarkers: (**a**) *TP53*, (**b**) *SIRT-1*, and (**c**) miR-34a.

Receiver operating characteristic (ROC) curve analysis was performed to evaluate the exploratory potential of the circulating biomarkers to differentiate between the studied groups. While all three markers achieved statistical significance, their individual discriminatory performance remained within a modest range. *SIRT-1* expression demonstrated the highest area under the curve (AUC) at 0.6987 (*p* < 0.0001), followed by *TP53* with an AUC of 0.6735 (*p* < 0.0001). miR-34a showed a more limited discriminatory capacity, with an AUC of 0.6000 (*p* = 0.0176). Although these results indicate a statistically significant ability to distinguish AF+ from AF− patients, when considering these findings alongside the risk analysis, circulating *SIRT-1* appears to be the primary marker with promising exploratory value, rather than definitive diagnostic utility. The ROC curves for all three biomarkers are presented in Figure 4. Internal validation using a bootstrap method was performed to assess model stability and correct for optimism bias. The optimism-corrected AUCs confirmed the stability of the ROC estimates. Specifically, the optimism-corrected AUC for *SIRT-1* was 0.700 (bootstrapped 95% CI: 0.620–0.775), and that for *TP53* was 0.675 (bootstrapped 95% CI: 0.591–0.746). Because the lower bounds of these 95% confidence intervals remained well above 0.50, the discriminatory capacity of both markers was confirmed to be statistically significant. The optimism-corrected estimate for miR-34a was 0.600, with a wider interval approaching the null threshold (bootstrapped 95% CI: 0.517–0.674).

Exploratory optimal cut-off expression level values, determined using the Youden index, were 0.029564 for *TP53*, 0.007867 for *SIRT-1*, and 0.045123 for miR-34a. Among the analyzed molecules, only circulating *TP53* was significantly associated with AF in the cut-off-based analysis. A peripheral *TP53* expression level above the optimal cut-off was associated with nearly threefold higher odds of AF (OR = 2.92, 95% CI: 1.61–5.28, *p* = 0.0006), whereas no significant associations were found for *SIRT-1* (OR = 1.26, 95% CI: 0.71–2.24, *p* = 0.50) or miR-34a (OR = 0.59, 95% CI: 0.13–2.55, *p* = 0.36). Given that these parameters were measured in peripheral blood, these cut-off values and corresponding odds should be interpreted as hypothesis-generating indicators of systemic dysregulation associated with AF.

We also constructed models adjusted for universally recognized cardiovascular cofounders: sex, BMI, hypertension, T2DM, and CAD (Table 7). Our comprehensive analysis demonstrated that the altered expression of *TP53* (β = 0.334, 95 CI [0.142, 0.527], *p* = 0.0008) and *SIRT-1* (β = −0.460, 95 CI [−0.750, −0.169], *p* = 0.0021) remained robustly and independently associated with AF. The association for miR-34a narrowly missed the traditional threshold for significance in this fully adjusted model (*p* = 0.052).

## 3. Discussion

In this study, we observed significant differences in peripheral molecular markers between patients with AF and individuals without AF. Specifically, AF was associated with higher *TP53* expression, higher miR-34a expression, and lower *SIRT-1* expression. Traditional metabolic parameters such as glucose and lipid fractions generally did not differ between groups, while the AF patients exhibited higher prevalence of nicotine use and elevated renal markers (urea and creatinine). This pattern suggests that AF is associated with systemic molecular alterations related to cellular stress, aging, inflammation, and renal function rather than overt differences in standard metabolic parameters.

### 3.1. The TP53/SIRT-1/miR-34a Loop in the Context of Cardiovascular Stress

The higher whole-blood *TP53* expression observed in patients with AF is consistent with the involvement of TP53/p53-related pathways in cellular stress responses, apoptosis, senescence, and inflammation. These biological processes have been implicated in cardiovascular aging and organ fibrosis [34]. Previous experimental and clinical studies suggest that p53 activation may contribute to adverse cardiac remodeling, the transition from adaptive hypertrophy to heart failure, and structural changes in cardiac tissue [35,36]. However, in the present study, *TP53* was assessed only at the mRNA level in whole blood. Thus, our findings cannot be considered direct evidence of increased p53 protein activity, p53 acetylation, or atrial tissue remodeling.

The lower whole-blood *SIRT-1* expression in the AF group is also of interest, because SIRT-1 is a NAD^+^-dependent deacetylase involved in cellular stress resistance, metabolic regulation, inflammation, and aging. *SIRT-1* can modulate p53-dependent signaling by deacetylating p53 and reducing its transcriptional activity [37,38]. Experimental studies have shown that SIRT-1-related pathways may protect against pathological myocardial remodeling by limiting oxidative stress and inflammatory responses [38]. Nevertheless, the present study measured *SIRT-1* mRNA expression rather than SIRT-1 protein abundance or deacetylase activity. Therefore, reduced SIRT-1 mRNA expression in whole blood should be interpreted as a peripheral biomarker alteration rather than the proof of reduced SIRT-1 functional activity in cardiac tissue.

Serum miR-34a levels were significantly higher in patients with AF. miR-34a is a well-described stress- and aging-related microRNA and has been linked to p53 signaling, SIRT-1 regulation, cellular senescence, endothelial dysfunction, and cardiovascular aging [39,40,41]. In experimental models, miR-34a may suppress *SIRT-1* expression and participate in feedback mechanisms involving p53 and *SIRT-1*. However, our data do not demonstrate such a coordinated mechanism in patients with AF. In the AF group, *TP53* expression correlated positively with *SIRT-1* expression, whereas *TP53* expression was not significantly correlated with serum miR-34a. Moreover, the inverse correlation between serum miR-34a and *SIRT-1* expression was observed only in individuals without AF and was not reproduced in AF patients.

Therefore, although *TP53*, miR-34a, and *SIRT-1* are biologically connected in experimental models, the correlation pattern observed in this study does not support the presence of a coordinated circulating TP53/miR-34a/SIRT-1 regulatory loop in AF. Although the negative association between miR-34a and *SIRT-1* observed in the control (AF−) group may be consistent with previously described regulatory relationships [36], this finding was not present in the AF group and should be interpreted cautiously as the markers were measured in different biological compartments.

Overall, our findings suggest that AF is accompanied by altered circulating systemic expression levels of *TP53*, miR-34a, and *SIRT-1*. These alterations may reflect broader systemic processes associated with AF, including aging, inflammation, oxidative stress, renal function, and cardiovascular comorbidity. This interpretation is supported by the observed associations of miR-34a with age, C-reactive protein, and estimated glomerular filtration rate in the AF group.

### 3.2. Potential Intersections with Remodeling Pathways

Previous research has linked the pathogenesis of AF to pathways of inflammation and extracellular matrix turnover, involving mediators such as matrix metalloproteinase-9, tumor necrosis factor-α, and myeloperoxidase [42,43,44]. While these molecules are known to play roles in atrial fibrosis and structural remodeling [42,44], it is critical to emphasize that none of these markers were measured in the present study. Consequently, our findings regarding *TP53*, miR-34a, and *SIRT-1* cannot be directly linked to these specific remodeling pathways within our cohort.

The observed correlations between serum miR-34a and systemic indicators, such as C-reactive protein and eGFR, suggest that the dysregulation of these molecules likely reflects a broader systemic stress response rather than a localized atrial signal. While experimental models suggest that p53 and SIRT-1 may intersect with inflammatory and fibrotic signaling [11,45], these remain theoretical associations in the context of our circulating data.

### 3.3. Compartment-Specific Interpretation of Circulating Markers

A key methodological issue is that *TP53* and *SIRT-1* were measured as mRNA expression in whole blood, whereas miR-34a was quantified in serum. These biological compartments differ substantially in cellular composition and molecular origin. Whole-blood mRNA largely reflects nucleated blood cells, particularly leukocyte subpopulations, while serum miRNAs may derive from multiple sources, including blood cells, platelets, endothelial cells, extracellular vesicles, damaged tissues, renal clearance-related mechanisms, and potentially cardiac tissue. Consequently, regulatory relationships inferred across these compartments should be interpreted with caution.

The present study did not include matched atrial tissue samples, isolated blood-cell populations, extracellular vesicle profiling, or tissue-specific validation. Therefore, the tissue source of the observed *TP53*, miR-34a, and *SIRT-1* alterations remains unknown. In particular, the data do not allow us to conclude that these circulating markers originate from atrial tissue or directly reflect atrial remodeling; thus, they should be interpreted as systemic molecular alterations associated with AF.

### 3.4. Clinical and Translational Implications

From a clinical perspective, the observed differences suggest that *TP53,* miR-34a, and *SIRT-1* may have potential as systemic biomarkers associated with AF. However, their diagnostic performance was modest, and only the *TP53* expression cut-off was significantly associated with AF in the Youden-derived odds-ratio analysis. Therefore, these markers should not be considered diagnostic tools at this stage. Their potential clinical value requires validation in larger, independent cohorts, preferably with multivariable adjustment, longitudinal follow-up, and comparison with established clinical risk factors.

Receiver operating characteristic analysis demonstrated that all three biomarkers—*TP53, SIRT-1*, and miR-34a—showed statistically significant capacity to differentiate AF patients from controls. *SIRT-1* exhibited the highest discriminatory power, while *TP53* demonstrated the strongest clinical association in cut-off analysis. This suggests that these molecules could potentially serve as exploratory components of multimarker panels for identifying individuals at higher risk for AF development or progression [11]. Interestingly, we observed specific correlations between these circulating markers and metabolic parameters, such as the positive associations between *SIRT-1* and cholesterol and triglycerides in the AF+ group. These findings suggest a potential interaction between systemic metabolic regulation and cellular stress signaling, which warrants further investigation into how metabolic stress influences microRNA and mRNA expression in the context of cardiovascular disease [46].

Furthermore, the observation of higher urea and creatinine levels in AF+ patients, alongside a negative correlation between miR-34a and eGFR, underscores the potential importance of renal function in the systemic molecular environment of AF. Renal impairment may amplify the systemic inflammatory and oxidative stress signals that are often observed in patients with AF [4,42]. Given that these markers were measured in peripheral blood, they reflect systemic molecular alterations associated with the arrhythmia rather than localized atrial molecular remodeling.

### 3.5. Limitations

This study has several limitations that should be considered when interpreting the findings. First, the modest sample size limits the statistical power and generalizability, and replication in larger, independent populations is necessary to confirm the observed associations. In addition, the duration of AF was not precisely documented, which may have affected the interpretation of biomarker changes in relation to the burden and progression of the disease.

Second, the analyses were performed exclusively on peripheral blood samples, with mRNA measured in whole blood and miRNA in serum. Although these specimens provide a non-invasive and clinically translatable approach, circulating biomarkers may reflect broader systemic processes, including inflammation and cardiorenal interactions, rather than the local molecular environment of the atrial myocardium. Therefore, our conclusions regarding atrial structural and electrical remodeling should be regarded as hypothesis-generating. Studies using matched peripheral blood and atrial tissue samples are needed to determine whether systemic dysregulation of the TP53/miR-34a/SIRT-1 axis parallels local atrial tissue expression.

Third, despite the prospective nature of the study, the associative character of the analysis precludes definitive conclusions concerning causality or temporal dynamics between biomarker changes and AF onset, progression, or recurrence. Furthermore, the observed positive correlation between *TP53* and *SIRT-1* in AF+ patients contradicts the canonical miR-34a-mediated inhibitory loop, suggesting that these molecules may not function as a coordinated regulatory axis in the circulation.

Fourth, there is potential confounding from limited data on comorbidities, antiarrhythmic and metabolic therapies, inflammatory status, and other factors; although adjustments were made for known covariates, unmeasured confounding cannot be excluded.

Fifth, our study did not include an independent external validation cohort, nor did we perform internal cross-validation. Because the optimal cut-off values and their corresponding odds ratios were derived from and tested on the same cohort, there is an inherent risk of overestimating their diagnostic performance. Given the relatively modest sample size, internal data partitioning (i.e., split-sample validation) would have resulted in underpowered subsets, potentially compromising the reliability of the validation. Therefore, our ROC analyses and cut-off values should be interpreted strictly as exploratory. Large-scale, multicenter external validation studies are essential to confirm the true diagnostic accuracy and clinical utility of this molecular profile before these biomarkers can be considered for clinical translation.

Finally, the study population’s ethnic and geographic characteristics may limit extrapolation to broader or more diverse populations. Further studies incorporating matched atrial tissue and peripheral blood samples, protein-level analyses, and functional assays are needed to determine whether these peripheral alterations are mechanistically involved in the pathophysiology of AF or serve primarily as systemic biomarkers associated with the disease.

## 4. Materials and Methods

### 4.1. Characteristics of Patients

In total, 189 adult patients aged 62.69 ± 13.85 years (including 58 women and 131 men) who were hospitalized between January 2024 and April 2024 for various internal diseases at the Department of Electrocardiology at the Medical University of Lodz were included in this observational case–control study. The study was performed under the guidelines of the Helsinki Declaration for human research and approved by the Bioethics Committee of Medical University of Lodz (approval number RNN/01/24/KE).

Participants were eligible for inclusion in the study if they met the following criteria: age over 18 years, provision of written informed consent to participate in the study, preserved verbal and logical contact enabling reliable communication, and confirmed diagnosis of AF (AF+ group). Participants included in the control group (AF− group) had no documented history of AF episodes based on medical history and available diagnostic examinations. Exclusion criteria included age under 18 years, lack of informed written consent to participate in the study, age-impaired verbal and logical communication with the patient, presence of secondary causes of AF (including alcohol and other substance use, hyperthyroidism and hypothyroidism, signs of acute infection, significant valvular heart disease, hypertrophic and dilated cardiomyopathy, tachycardia-bradycardia syndrome, previous heart surgery), presence of chronic inflammatory diseases and active and past malignancy, presence of systemic disease with cardiac involvement (sarcoidosis, amyloidosis, hemochromatosis), pregnancy, and likelihood of paroxysmal AF based on the patient’s history, despite the lack of confirmation of this arrhythmia in diagnostic tests.

The enrolled patients had their medical history taken and underwent a physical examination. Blood pressure (BP)—including systolic blood pressure (SBP) and diastolic blood pressure (DBP)—and anthropometric measurements (body weight, height, waist (WC) and hip circumference (HC)) were recorded and used to calculate the body mass index (BMI) and waist–hip ratio (WHR). Next, blood samples were collected to determine fasting plasma glucose (FPG); glycated hemoglobin level (HbA1c); total cholesterol (T-CH); LDL cholesterol (LDL-CH); HDL cholesterol (HDL-CH); triglycerides (TG); total bilirubin; uric acid; urea; C-reactive protein (CRP); creatinine concentrations; liver enzymes, including alanine aminotransferase (ALT); asparagine (AST); and gamma-glutamyltransferase (GGTP) activity. The estimated glomerular filtration rate (eGFR) was calculated based on the Modification of Diet in Renal Disease equations. Then each patient underwent a standard ECG recording (including a 24 h Holter recording) with thorough analysis, confirming the presence of AF or excluding cardiac arrhythmias. Transthoracic echocardiography (TTE) was also performed to assess the left ventricular ejection fraction (EF).

Subsequently, the participants were assigned to two groups: patients with (AF+) and without AF (AF−). The groups were then compared to assess differences, especially in circulating levels of TP53, miR-34a, and SIRT-1. In addition, the diagnostic utility of these biomarkers in the detection of AF was evaluated.

### 4.2. Analysis of mRNA Expression

Total RNA was isolated from the blood samples using a Total RNA Mini Kit (A&A Biotechnology, Gdynia, Poland). The concentration and purity of the isolated RNA were subsequently determined spectrophotometrically using a Nanodrop 2000 (Thermo Fisher Scientific Inc., Waltham, MA, USA). One microgram (1 μg) of the total RNA was converted to complementary DNA (cDNA) via reverse transcription using the High-Capacity cDNA Reverse Transcription Kit (Thermo Fisher Scientific Inc., Waltham, MA, USA), strictly following the manufacturer’s established protocols. The resultant cDNA served as the template for quantitative real-time PCR (qRT-PCR) amplification. The reaction utilized the TaqMan assay (Life Technologies, Carlsbad, CA, USA) and the TaqMan Universal Master Mix (Life Technologies, Carlsbad, CA, USA). Each sample was analyzed in duplicate. Target gene: TP53 (ID: Hs01034249_m1) and SIRT-1 (Hs01009006_m1). Reference genes: GAPDH (ID: Hs99999905_m1) were employed as the endogenous control for normalization. The expression levels of TP53 and SIRT-1 are reported as median and upper and lower quartiles. 

For analysis of miRNA expression, total RNA was isolated from the serum samples using an RNA Isolation Kit Plasma/Serum (BioVendor, Brno, Czech Republic). The expression level of the selected miR-34a was quantified using RT-qPCR. First, 2 µL of total RNA was reverse transcribed in a 15 µL reaction volume using TaqMan MicroRNA Reverse Transcription kit (Applied Biosystems^TM^, ThermoFisher Scientific, Waltham, MA, USA), according to the manufacturer’s protocol. The RT reaction was conducted using a T100 Thermal Cycler (BioRad, Hercules, CA, USA). The resulting cDNA was used as the template for qPCR amplification. The 10 µL qPCR reaction mixture comprised 2.5 µL of diluted cDNA, 5 µL of TaqMan^TM^ Universal Master Mix II, no UNG (Applied Biosystems^TM^, ThermoFisher Scientific, Waltham, MA, USA), 0.5 µL of the specific TaqMan^TM^ MicroRNA Assay (Applied Biosystems^TM^, ThermoFisher Scientific, Waltham, MA, USA) for the target miR-34a (ID: 478047_miR), and 2 µL of nuclease-free water. Each sample was processed into independent replicates. miR-186 (477940_mir) and miR-103 (478253_mir) were utilized as endogenous reference genes for normalization. The expression level of miR-34a is presented as median and upper and lower quartiles.

### 4.3. Statistical Analysis

Anthropometric measures, biochemical characteristics, miR levels, and gene expression across the groups are summarized using medians and the corresponding lower and upper quartiles, given the non-normal distribution of variables. The Shapiro–Wilk *t* test confirmed that the variable distributions deviated from normality. Consequently, the Mann–Whitney U test was employed to assess differences between two independent groups for continuous variables. A Chi-square test was used to compare the group ratios for categorical variables; i.e., sex ratio and comorbidities. The relationship between miR or gene expression and other parameters was determined using the Spearman non-parametric correlation coefficient. To account for the increased risk of type I errors arising from multiple comparisons, the Benjamini–Hochberg procedure was applied to all correlation analyses. The False Discovery Rate (FDR) was controlled at a threshold of 0.05. Only correlations that remained significant after FDR adjustment (q < 0.05) were considered statistically significant and included in the final interpretation. The diagnostic and prognostic usefulness of TP53, mir-34a, and SIRT-1 was evaluated using receiver operating characteristic (ROC) curve analysis to assess their ability to differentiate between AF+ and AF− group. A higher area under the ROC curve (AUC) indicates better discriminatory performance. To assess the stability of the diagnostic performance and correct for potential overfitting bias from deriving cut-offs within the same cohort, internal validation of the ROC curves was performed. Bootstrapping was utilized to generate internally validated 95% Confidence Intervals for the AUC estimates. Optimal cut-off values for the analyzed biomarkers were determined using the Youden index, and their associations with atrial fibrillation were assessed by calculating odds ratios (ORs) with 95% confidence intervals using chi-square tests with Yates’ or Fisher’s correction, as appropriate. All statistical calculations were executed using the GraphPad Prism 8.0 (San Diego, CA, USA) software package. A *p*-value < 0.05 was defined as the threshold for statistical significance.

## 5. Conclusions

AF was associated with higher expression of circulating *TP53*, miR-34a, and lower *SIRT-1*. These findings suggest systemic molecular alterations associated with AF and may indicate their potential as exploratory biomarkers, particularly *TP53* and *SIRT-1*. However, because the markers were measured in different biological compartments and did not show a consistent inter-marker correlation pattern, the present data do not indicate the dysregulation of a coordinated TP53/miR-34a/SIRT-1 regulatory axis. Moreover, in the absence of matched atrial tissue and lack of functional protein-level assays, the results should not be interpreted as direct evidence of atrial remodeling. Future studies should combine circulating and atrial tissue compartment measurements with assessments of p53 acetylation, *SIRT-1* activity, inflammatory mediators, and longitudinal clinical outcomes.

## Figures and Tables

**Table 1:** The distribution of comorbid diseases and related medications in AF+ and AF− groups and types of AF.

	AF− Group (n = 95)	AF+ Group (n = 94)	*p* *
**Comorbidity:**			
HA [%]	72.63	79.79	0.2482
T2DM [%]	29.47	39.36	0.1525
CAD [%]	29.47	24.47	0.4382
Dyslipidemia [%]	47.37	50	0.7174
Stroke [%]	6.32	10.64	0.2858
Nicotine addiction [%]	6.32	19.15	**0.0081**
Hypothyroidism [%]	4.21	5.32	0.7810
Hyperthyroidism [%]	10.53	12.77	0.6312
CHF [%]	32.63	45.74	0.0648
CKD [%]	8.42	15.96	0.1131
COPD [%]	7.37	9.57	0.5859
MASLD [%]	51.59	60.64	0.2096
**Type of AF:**			
Paroxysmal	0	31	**<0.0001**
Persistent	0	49	**<0.0001**
Permanent	0	14	**<0.0001**
**Medications:**			
Antiarrhythmic drugs	67	82	0.0049
Oral anticoagulant	15	85	**<0.0001**
ACE inhibitor/sartan	70	71	0.7705
Diuretic	37	50	0.0495
Statin	53	58	0.4091
Flosin	26	25	0.9048

AF—atrial fibrillation; CAD—coronary artery disease; CHF—chronic heart failure; CKD—chronic kidney disease; COPD—chronic obstructive lung disease; HA—hypertension; MASLD—metabolic dysfunction-associated steatotic liver disease; T2DM—type 2 diabetes. * *p*-value was assessed using the Chi^2^ test. Data are expressed as percentages. The bolded results indicate statistically significant differences.

**Table 2:** The distribution of age, sex, anthropometric parameters, blood pressure, and ejection fraction in AF+ and AF− groups.

Parameter	AF− Group (n = 95)	AF+ (n = 94)	*p* *
Sex [% of F]	35.79	25.53	0.1263
Age [years]	63 [46; 72]	67 [60; 72]	0.0595
Height [cm]	170 [168; 178]	174 [166; 180]	0.4497
Body weight [kg]	82 [72; 95]	85.5 [77; 100]	0.0682
BMI [kg/m^2^]	28.13 [24.61; 31.56]	29.15 [26.34; 31.86]	0.0807
WC [cm]	100 [90; 108]	104 [93.75; 110.3]	0.1035
HC [cm]	104 [98; 112]	105.5 [102; 112]	0.2838
WHR	0.94 [0.86; 1.00]	0.96 [0.8875; 1.020]	0.1947
SBP [mmHg]	127 [112; 140]	130 [115; 140]	0.6361
DBP [mmHg]	78 [70; 82]	80 [70; 83.25]	0.9502
EF [%]	56.00 [45; 60]	55 [43; 60]	0.4901

BMI—body mass index; DBP—diastolic blood pressure; EF—ejection fraction; HC—hip circumference; SBP—systolic blood pressure; WC—waist circumference; WHR—waist to hip ratio. * *p*-value was assessed using the Mann–Whitney U test. Data are expressed as medians (quartile 1; quartile 3).

**Table 3:** Biochemical characteristics of the study population.

Parameter	AF− Group (n = 95)	AF+ Group (n = 94)	*p* *
Urea [mmol/L]	6.12 [4.85; 7.62]	6.805 [5.25; 8.743]	**0.0264**
Creatinine [µmol/L]	84.30 [68.90; 101.2]	91.20 [78.15; 108.5]	**0.0235**
eGFR [ml/min/1.73 m^2^]	75.70 [55.50; 93.60]	69.40 [56.78; 89.95]	0.0958
FPG [mmol/L]	5.68 [5.19; 6.54]	5.745 [5.23; 6.52]	0.9667
HbA1c [%]	5.7 [5.4; 6.1]	5.7 [5.4; 6.1]	0.6965
Uric acid [µmol/L]	318.4 [258.5; 403]	337.3 [299.6; 395.6]	0.1088
T-CH [mmol/L]	4.6 [3.57; 5.35]	4.305 [3.533; 5.305]	0.8469
LDL-CH [mmol/L]	2.7 [1.88; 3.2]	2.44 [1.98; 3.12]	0.2723
HDL-CH [mmol/L]	0.99 [0.78; 1.45]	1.045 [0.86; 1.385]	0.5072
TG [mmol/L]	1.67 [1.01; 2.37]	1.52 [0.97; 2.183]	0.3621
ALT [U/L]	22.65 [16.6; 32.3]	23.35 [18.08; 30.18]	0.8562
AST [U/L]	25.1 [21.2; 30.8]	25.85 [21.45; 31.55]	0.7376
GGTP [U/L]	29.2 [20.20; 45.00]	33.35 [24.25; 46.73]	0.0943
Total bilirubin [µmol/L]	12.8 [9.4; 17.50]	13.80 [10.38; 17.90]	0.1368
CRP [mg/L]	2 [0.8; 4.7]	2.3 [0.975; 4.650]	0.5463

ALT—alanine aminotransferase; AST—asparagine aminotransferase; CRP—C-reactive protein; eGFR—estimated glomerular filtration rate; FPG—fasting plasma glucose; GGTP—gamma-glutamyltransferase; HbA1c—glycated hemoglobin level; HDL-CH—HDL cholesterol; LDL-CH—LDL cholesterol; T-CH—total cholesterol; TG—triglycerides. * *p*-value was assessed using the Mann–Whitney U test. Data are expressed as medians (quartile 1; quartile 3). The bolded results indicate statistically significant differences.

**Table 4:** Univariate correlations of tumor protein *TP53* expression and clinical parameters of AF patients and AF− (control) groups.

Variable	AF− Group (n = 95)		AF+ Group (n = 94)	
	*Rho* **	*p* *	*Rho* **	*p* *
Age [years]	−0.0764	0.4613	−0.1162	0.2643
Body weight [kg]	0.2630	0.0100	0.0993	0.3407
BMI [kg/m^2^]	0.2417	0.0182	0.0801	0.4427
WC [cm]	0.1395	0.1773	0.0551	0.5977
HC [cm]	0.2007	0.0510	0.0776	0.4567
WHR	0.0179	0.8628	−0.0200	0.8481
SBP [mmHg]	−0.1398	0.1763	−0.0278	0.7900
DBP [mmHg]	−0.0828	0.4245	0.0660	0.5269
EF [%]	0.0670	0.5184	−0.0537	0.6068
Urea [mmol/L]	−0.0528	0.6108	0.1128	0.2786
Creatinine [µmol/L]	−0.0865	0.4043	0.1209	0.2455
eGFR [mL/min/1.73 m^2^]	0.1253	0.2263	−0.0285	0.7844
FPG [mmol/L]	0.1296	0.2103	−0.0548	0.5992
HbA1c [%]	0.1297	0.2100	0.0192	0.8542
Uric acid [µmol/L]	−0.0234	0.8215	−0.1364	0.1898
T-CH [mmol/L]	−0.0096	0.9259	0.1586	0.1266
LDL-CH [mmol/L]	0.0432	0.6771	0.0480	0.6471
HDL-CH [mmol/L]	−0.0977	0.3462	0.1995	0.0537
TG [mmol/L]	0.0995	0.3369	0.0926	0.3743
ALT [U/L]	0.0407	0.6968	0.1561	0.1329
AST [U/L]	0.0207	0.8415	0.0898	0.3889
GGTP [U/L]	0.1913	0.0631	−0.1054	0.3119
Total bilirubin [µmol/L]	−0.0885	0.3934	0.0846	0.4172
CRP [mg/L]	0.0867	0.4030	−0.0155	0.8818
miR-34a	−0.0377	0.7161	−0.0884	0.3965
SIRT-1	0.0660	0.5250	0.3609	**<0.001**

ALT—alanine aminotransferase; AST—asparagine aminotransferase; BMI—body mass index; CRP—C-reactive protein; DBP—diastolic blood pressure; EF—ejection fraction; eGFR—estimated glomerular filtration rate; FPG—fasting plasma glucose; GGTP—gamma-glutamyltransferase; HbA1c—glycated hemoglobin level; HC—hip circumference; HDL-CH—HDL cholesterol; LDL-CH—LDL cholesterol; SBP—systolic blood pressure; T-CH—total cholesterol; TG—triglycerides; WC—waist circumference; WHR—waist to hip ratio. Significant correlations assessed using Spearman’s rank correlation analysis; ** *Rho*, Spearman’s correlation coefficient; * *p*, *p*-value. The bolded results indicate statistically significant differences.

**Table 5:** Univariate correlations of *SIRT-1* expression level and clinical parameters of AF patients and control individuals.

Variable	AF− Group (n = 95)		AF+ Group (n = 94)	
	*Rho* **	*p* *	*Rho* **	*p* *
Age [years]	0.0152	0.8837	−0.1742	0.0930
Body mass [kg]	0.0799	0.4413	0.0742	0.4768
BMI [kg/m^2^]	0.0883	0.3945	0.0795	0.4458
WC [cm]	0.0490	0.6366	0.0680	0.5146
HC [cm]	0.0499	0.6306	0.0509	0.6259
WHR	0.0240	0.8167	0.0074	0.9435
SBP [mmHg]	0.0127	0.9025	0.0036	0.9724
DBP [mmHg]	−0.0634	0.5413	0.0664	0.5246
EF [%]	0.1440	0.1637	0.0518	0.6194
Urea [mmol/L]	−0.0356	0.7312	−0.0305	0.7703
Creatinine [µmol/L]	−0.0520	0.6163	−0.0034	0.9734
eGFR [mL/min/1.73 m^2^]	−0.0211	0.8391	0.1600	0.1232
FPG [mmol/L]	−0.0154	0.8821	−0.0121	0.9072
HbA1c [%]	−0.0760	0.4635	−0.0519	0.6193
Uric acid [µmol/L]	0.0505	0.6266	−0.1816	0.0797
T-CH [mmol/L]	0.1782	0.0839	0.2152	0.0371
LDL-CH [mmol/L]	0.1455	0.1591	0.0816	0.4366
HDL-CH [mmol/L]	0.2116	0.0394	0.1166	0.2628
TG [mmol/L]	−0.1372	0.1846	0.2601	0.0113
ALT [U/L]	0.0590	0.5717	0.1065	0.3066
AST [U/L]	0.05645	0.5868	−0.1007	0.3337
GGTP [U/L]	−0.1832	0.07555	0.0369	0.7236
Total bilirubin [µmol/L]	−0.1501	0.1464	0.0683	0.5126
CRP [mg/L]	0.0025	0.9806	−0.0530	0.6118
TP53	0.0660	0.5250	0.3609	**0.0003**
miR-34a	−0.2850	0.0051	−0.0113	0.9138

ALT—alanine aminotransferase; AST—asparagine aminotransferase; BMI—body mass index; CRP—C-reactive protein; DBP—diastolic blood pressure; EF—ejection fraction; eGFR—estimated glomerular filtration rate; FPG—fasting plasma glucose; GGTP—gamma-glutamyltransferase; HbA1c—glycated hemoglobin level; HC—hip circumference; HDL-CH—HDL cholesterol; LDL-CH—LDL cholesterol; SBP—systolic blood pressure; T-CH—total cholesterol; TG—triglycerides; WC—waist circumference; WHR—waist to hip ratio. Significant correlations assessed using Spearman’s rank correlation analysis; ** *Rho*, Spearman’s correlation coefficient; * *p*, *p*-value. The bolded results indicate statistically significant differences.

**Table 6:** Univariate correlations of serum level of miR-34a expression and clinical parameters in AF+ and AF− (control) groups.

Variable	AF− Group (n = 95)		AF+ Group (n = 94)	
	*Rho* **	*p* *	*Rho* **	*p* *
Age [years]	0.3999	**<0.001**	0.2653	0.0097
Body mass [kg]	−0.1152	0.2659	0.0230	0.8253
BMI [kg/m^2^]	−0.0087	0.9329	0.1599	0.1236
WC [cm]	0.0459	0.6585	0.1351	0.1941
HC [cm]	−0.0539	0.6037	0.0380	0.7154
WHR	0.0723	0.4856	0.1247	0.2309
SBP [mmHg]	0.1352	0.1912	−0.0701	0.5016
DBP [mmHg]	−0.0077	0.9404	−0.0377	0.7177
EF [%]	−0.2690	**0.0083**	−0.1177	0.2584
Urea [mmol/L]	0.0405	0.6961	0.1684	0.1045
Creatinine [µmol/L]	0.0184	0.8592	0.1378	0.1852
eGFR [mL/min/1.73 m^2^]	−0.1951	0.0581	−0.2862	0.0051
FPG [mmol/L]	0.1240	0.2308	0.1974	0.0565
HbA1c [%]	0.1050	0.3108	0.1197	0.2504
Uric acid [µmol/L]	−0.1916	0.0628	0.1521	0.1433
T-CH [mmol/L]	−0.2093	0.0417	−0.1219	0.2416
LDL-CH [mmol/L]	−0.2700	**0.0081**	−0.0612	0.5597
HDL-CH [mmol/L]	−0.0881	0.3957	−0.0033	0.9740
TG [mmol/L]	0.2951	**0.0036**	−0.1139	0.2739
ALT [U/L]	−0.1469	0.1574	−0.0625	0.5494
AST [U/L]	−0.0184	0.8588	0.0571	0.5843
GGTP [U/L]	0.0921	0.3745	0.0211	0.8400
Total bilirubin [µmol/L]	0.0758	0.4651	−0.0191	0.8548
CRP [mg/L]	−0.0681	0.5118	0.2099	0.0422
TP53	−0.0377	0.7161	−0.0884	0.3965
SIRT-1	−0.2850	**0.0051**	−0.0113	0.9138

ALT—alanine aminotransferase; AST—asparagine aminotransferase; BMI—body mass index; CRP—C-reactive protein; DBP—diastolic blood pressure; EF—ejection fraction; eGFR—estimated glomerular filtration rate; FPG—fasting plasma glucose; GGTP—gamma-glutamyltransferase; HbA1c—glycated hemoglobin level; HC—hip circumference; HDL-CH—HDL cholesterol; LDL-CH—LDL cholesterol; SBP—systolic blood pressure; T-CH—total cholesterol; TG—triglycerides; WC—waist circumference; WHR—waist to hip ratio. Significant correlations assessed using Spearman’s rank correlation analysis; ** *Rho*, Spearman’s correlation coefficient; * *p*, *p*-value. The bolded results indicate statistically significant differences.

**Table 7:** Multivariable linear regression models evaluating the association of AF with expression level of miR-34a, *TP53*, and *SIRT-1*, adjusted for sex, BMI, hypertension, T2DM, and ischemic heart disease.

Variable	AF β Coefficient	95 CI Lower	95% CI Upper	*p* for AF
miR-34a	0.6822	−0.0068	1.3713	0.0523
*TP53*	0.3343	0.1418	0.5268	0.0008
*SIRT-1*	−0.4598	−0.7504	−0.1692	0.0021

## Data Availability

The datasets used and/or analyzed during the current study are available from the corresponding author on reasonable request. The data are not publicly available due to privacy.

## Abbreviations

The following abbreviations are used in this manuscript:

AF	Atrial fibrillation
ALT	Alanine aminotransferase
AST	Asparagine aminotransferase
AUC	Area under the ROC curve
BMI	Body mass index
BP	Blood pressure
CAD	Coronary artery disease
CHF	Chronic heart failure
CKD	Chronic kidney disease
COPD	Chronic obstructive lung disease
CRP	C-reactive protein
DBP	Diastolic blood pressure
ECG	Electrocardiography
eGFR	Estimated glomerular filtration rate
EF	Left ventricular ejection fraction
FPG	Fasting plasma glucose
GGTP	Gamma-glutamyltransferase
HA	Hypertension
HbA1c	Glycated hemoglobin level
HC	Hip circumference
HDL-CH	HDL cholesterol
LDL-CH	LDL cholesterol
MASLD	Metabolic dysfunction-associated steatotic liver disease
ORs	Odds ratios
SBP	Systolic blood pressure
T2DM	type 2 diabetes
T-CH	Total cholesterol
TG	Triglycerides
TTE	Transthoracic echocardiography
WC	Waist circumference
WHR	Waist–hip ratio
